# Excitation/Inhibition balance relates to cognitive function and gene expression in temporal lobe epilepsy: a high density EEG assessment with aperiodic exponent

**DOI:** 10.1093/braincomms/fcae231

**Published:** 2024-07-08

**Authors:** Gian Marco Duma, Simone Cuozzo, Luc Wilson, Alberto Danieli, Paolo Bonanni, Giovanni Pellegrino

**Affiliations:** Scientific Institute IRCCS E.Medea, Epilepsy and Clinical Neurophysiology Unit, 31015, Conegliano, Italy; Scientific Institute IRCCS E.Medea, Epilepsy and Clinical Neurophysiology Unit, 31015, Conegliano, Italy; McConnell Brain Imaging Centre, Montreal Neurological Institute, McGill University, Montreal, QC H3A 2B4, Canada; Scientific Institute IRCCS E.Medea, Epilepsy and Clinical Neurophysiology Unit, 31015, Conegliano, Italy; Scientific Institute IRCCS E.Medea, Epilepsy and Clinical Neurophysiology Unit, 31015, Conegliano, Italy; Department of Clinical Neurological Sciences, Schulich School of Medicine and Dentistry, Western University, London N6A5C1, Canada

**Keywords:** excitation/inhibition balance, aperiodic exponent, temporal lobe epilepsy, genetic expression, memory functioning

## Abstract

Patients with epilepsy are characterized by a dysregulation of excitation/inhibition balance (E/I). The assessment of E/I may inform clinicians during the diagnosis and therapy management, even though it is rarely performed. An accessible measure of the E/I of the brain represents a clinically relevant feature. Here, we exploited the exponent of the aperiodic component of the power spectrum of the electroencephalography (EEG) signal, as a non-invasive and cost-effective proxy of the E/I balance. We recorded resting-state activity with high-density EEG from 67 patients with temporal lobe epilepsy and 35 controls. We extracted the exponent of the aperiodic fit of the power spectrum from source-reconstructed EEG and tested differences between patients with epilepsy and controls. Spearman’s correlation was performed between the exponent and clinical variables (age of onset, epilepsy duration and neuropsychology) and cortical expression of epilepsy-related genes derived from the Allen Human Brain Atlas. Patients with temporal lobe epilepsy showed a significantly larger exponent, corresponding to inhibition-directed E/I balance, in bilateral frontal and temporal regions. Lower E/I in the left entorhinal and bilateral dorsolateral prefrontal cortices corresponded to a lower performance of short-term verbal memory. Limited to patients with temporal lobe epilepsy, we detected a significant correlation between the exponent and the cortical expression of *GABRA1*, *GRIN2A*, *GABRD*, *GABRG2*, *KCNA2* and *PDYN* genes. EEG aperiodic exponent maps the E/I balance non-invasively in patients with epilepsy and reveals a close relationship between altered E/I patterns, cognition and genetics.

## Teaser

Duma *et al*., reported alteration of excitation/inhibition balance in patients with temporal lobe epilepsy (TLE). The excitation/inhibition balance was investigated non-invasively using as a proxy the aperiodic exponent of the electroencephalography (EEG) power spectrum. The aperiodic exponent correlated with memory functioning and cortical expression of genes involved in the excitation/inhibition regulation.

## Graphical Abstract

**Graphical Abstract:**
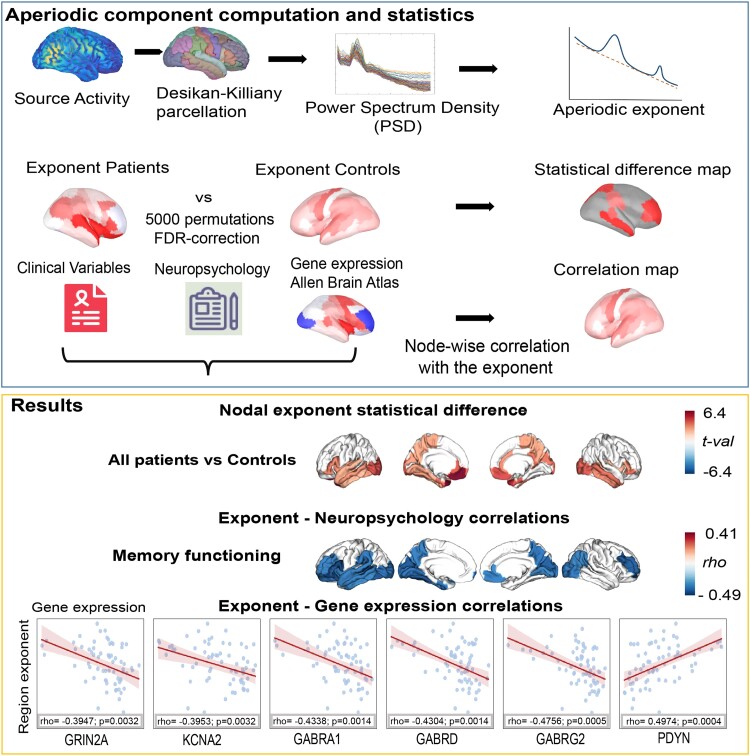

## Introduction

Epilepsy is a disorder characterized by a dysregulation of the excitation/inhibition balance (E/I) as suggested by converging evidence ranging from genetics, to proteomics expression, to clinical presentation.^1-4^ Altered E/I balance translates into an increased risk of recurrent epilepsy seizures.^5-7^ Hence, pharmacological treatments aim to reduce the risk of seizures by interfering with E/I through several mechanisms of action.^8-10^ Despite its biological and clinical relevance, E/I balance is rarely investigated directly in epilepsy, most likely because its estimation is difficult and time-consuming. Fortunately, recent developments offer a streamlined assessment of E/I balance, leveraging properties of spontaneous electromagnetic signals which can be easily measured with magneto- or electroencephalography (EEG) at high spatial and temporal resolutions, providing extensive brain coverage with potential translations in a clinical setting.^11-14,15^ Amongst multiple signal features, the exponent of the aperiodic component of the M/EEG signal power spectrum provides information related to system E/I balance.^16-18^

In this study, we investigated E/I balance in patients with temporal lobe epilepsy (TLE) in comparison to healthy subjects, by measuring the aperiodic exponent obtained from cortical reconstructed high-density EEG signals at rest. From a clinical perspective, TLE is of particular interest as it is one of the most prevalent and homogeneous manifestations of focal epilepsy.^19,20^ Considering the network nature of this neurological disorder, we hypothesized to detect more prominent E/I alterations in brain regions related to seizure onset, namely temporal lobe, as well as interconnected regions. Moreover, we expected to detect patterns of E/I changes influenced by the laterality of the epileptic focus. We further hypothesized a relation between E/I balance and clinical variables as well as an influence of antiseizure medications (ASMs) due to their known interaction with this property.^21,22^ Disrupted E/I balance may impact synaptic plasticity which represents one of the scaffolding mechanisms allowing a system to respond efficiently to environmental stimuli.^23,24^ Therefore, altered E/I balance may be connected to impairments detected in the TLE population in different cognitive domains and linked to functional network disruption at multiple spatial scales.^25-28^ As such, we would expect to detect a relationship between the aperiodic exponent and neuropsychological profiles in patients with TLE. Importantly, E/I balance is a multifactorial byproduct of neuronal activity, influenced by environmental factors as well as gene expression of channels and receptors.^29,30^ Numerous mutations of gene-regulating gamma-aminobutyric acid (GABA) receptors, as well as sodium-potassium channels, have been suggested as etiopathogenic causes of epilepsy.^31-34^ The recent release of open transcriptomics datasets such as the Allen Human Brain Atlas (AHBA) has offered opportunities to explore how gene expression patterns in the brain reflect macro-scale neurofunctional findings.^35,36^ Functional-genetic information integration can shed light on the micro- to macro-scale pathophysiological mechanisms underlying epilepsy. Here, we exploited transcriptomic data in order to connect epilepsy-related genes with E/I balance. Overall, the present study investigates the feasibility of the aperiodic component of the power spectrum density (PSD) via high-density electroencephalography (hdEEG) as a non-invasive measure of E/I balance, linking it to clinical, neuropsychological and genetic factors to elucidate the neural mechanisms underlying brain dynamics and cognitive alteration in the TLE.

## Materials and methods

### Participants

We enrolled patients with temporal lobe epilepsy who underwent hdEEG for clinical evaluation between 2018 and 2022 at the Epilepsy and Clinical Neurophysiology Unit, IRCCS Eugenio Medea in Conegliano (Italy) and a group of healthy controls (HCs). The diagnosis of temporal lobe epilepsy was established according to ILAE guidelines.^37^ The clinical workflow included Video EEG (32 channels) monitoring, brain magnetic resonance imaging (MRI) and positron emission tomography as an adjunctive investigation in selected patients. Seventy-two cases were retrospectively screened and five patients were excluded due to previous brain surgery or invasive investigation, resulting in a final sample size of 67 [mean age = 37.18 (SD = 18.04); 24 females; 30 left-TLE; 17 right-TLE; 20 bilateral-TLE]. The 89.5% of patients were drug-resistant. Patients’ demographic and clinical characteristics are provided in Table 1. The control group was composed of 35 healthy participants with no prior history of neurological or psychiatric disorders [mean age = 34.92 (SD = 9.22); 25 females]. The study protocol was conducted according to the Declaration of Helsinki and approved by the local ethical committee. All participants provided written informed consent to participate in the study.

**Table 1:** Sample demographic and clinical information

Patients with TLE	Mean ± Standard deviation
Age	40.93 ± 17.39
Age of onset	23.63 ± 17.50
Duration of epilepsy (years)	17.75 ± 18.03
Number of antiseizure medications	1.88 ± 1.03
**Antiseizure medications**	**Number**
ACT	1
AZM	2
BRV	7
CBZ	14
CLB	8
CZP	2
ESL	15
LCM	15
LEV	11
LTG	6
OXC	7
PB	2
PER	15
VPA	12
ZNS	1
NO-ASMs	5
**MRI**	
**Mesial**	**Number**
HS	14
DNET	1
UKN	10
Amygdala enlargement	6
**Anterior (temporal pole)**	
FCD	11
Encephalocele	2
Gliosis	2
**Anterior + mesial**	
FCD + HS	5
Developmental venous anomaly	1
**Negative MRI**	15

Demographic and clinical features of the patients. MRI findings are reported by sublobar localization. The continuous variables are reported as mean ± SD. Antiseizure medications abbreviations: ACT, acetazolamide; AZM, acetazolamide; BRV, brivaracetam; CBZ, barbamazepine; CLB, blobazam; CZP, blonazepam; ESL, eslicarbazepine; LCM, lacosamide; LEV, levetiracetam; LTG, lamotrigine; OXC, oxcarbazepine; PB, phenobarbital; PER, perampanel; VPA, valproic acid; ZNS, zonisamide; NO-ASMs, no pharmacological treatment. Abbreviation of MRI abnormalities: FCD, focal cortical dysplasia; HS, hippocampal sclerosis; DNET, hysembryoplastic neuroepithelial tumours; UKN, unknown.

### Neuropsychological assessment

Neuropsychological assessment focuses on memory, attention/executive functions and intelligence. Short-term memory and long-term memory functioning were assessed using the Digit Span,^38^ Corsi block tapping tests,^38^ Rey–Osterrieth Complex Figure Test (ROCFT)^39^ and Rey Auditory Verbal Learning Test (RAVLT).^40^ Attention and executive functions were evaluated using the Trail Making Test (TMT) (part A and B).^41^ Finally, we used the total intelligence quotient (IQ) of the WAIS-IV^42^ or WISC-IV^43^ scales as a measure of global intelligence. Table 2 shows the descriptive statistics of the neuropsychological scores.

**Table 2:** Neuropsychological scores

Test	Score (mean ± standard deviation)
Digit span	5.73 ± 1.06
Corsi block tapping test	4.90 ± 1.11
ROCFT—copy	30.81 ± 7.20
ROCFT—reproduction	15.03 ± 6.22
RAVLT—immediate	39.01 ± 9.75
RAVLT—delayed	7.17 ± 3.24
Total IQ	90.13 ± 19.62
TMT-A	34.47 ± 18.05
TMT-B	110.25 ± 76.84

### Resting-state EEG recording

Ten minutes of eyes-closed resting-state hdEEG was recorded with a 128-channel Micromed system while participants were sat comfortably on a chair in a silent room. The signal was sampled at 1024 Hz and referenced to the vertex. The impedance of the electrodes was kept <5 kΩ for each sensor.

### EEG preprocessing

Signal preprocessing was performed in EEGLAB 14.1.2b^44^ according to a pipeline validated in previous works.^26,45^ The preprocessing pipeline included the following steps: (a) resampling to 250 Hz; (b) bandpass-filter (0.1 to 45 Hz) with a Hamming windowed sinc finite impulse response filter (filter order = 8250); (c) epoching (1 s-long epochs resulting in 600 epochs per subject); (d) removal of epochs and channels containing interictal epileptiform discharges (IEDs) and artifacts; (e) removal of flat channels; (f) independent component analysis (40 independent components decomposition),^46^ using the Infomax algorithm^47^ as implemented in EEGLAB; (g) channel interpolation with spherical spline interpolation method^48^; (h) re-reference to average; (i) re-concatenation of the epochs obtaining a continuous signal.

In further detail, IEDs were identified by trained personnel (GMD, AD and PB). Epochs containing IEDs were purposely removed to assess intrinsic brain functional organization independently from epileptiform activity; automated bad-channel and artifact detection and removal was performed with the Trial-by-Trial plugin implemented in EEGLAB, with the following parameters: channels differential average amplitude 250 μV in more than 30% of the epochs. Epochs were removed if they contained IEDs, or included more than 10 bad channels. Flat channels were identified and removed with the Trimoutlier EEGLAB plug-in setting within the lower bound of 1 μV. We rejected an average of 46.80 ± 44.40 (SD) (average of 7.8% of rejected epochs) epochs and an average of 9.51 ± 4.68 (SD) (average of 23.7% of rejected components) components. After preprocessing, each subject had at least 6 min of artifact-free continuous signal.

### Cortical source modelling

For most of the TLE patients, the individual anatomic MRI for source imaging consisted of a T1 isotropic three-dimensional (3D) acquisition. For 12 patients and the control group, we used the MNI-ICBM152 default anatomy.^49^ The MRI was processed with the computational anatomy toolbox (CAT12),^50^ to obtain skin, skull and grey matter surfaces. Anatomical data were then imported in Brainstorm.^51^ The co-registration of the EEG electrodes with brain MRI was performed using Brainstorm, considering anatomical landmarks. Manual co-registration corrections were applied as needed. The cortical mesh was downsampled at 15 002 vertices and the three boundary element model (BEM) surfaces were reconstructed (inner skull, outer skull and head). We computed the forward model with the three-shell BEM approach, setting the tissue connectivity of the brain, skull and skin to 0.33, 0.165, 0.33 S/m; (ratio: 1/20), respectively. This was achieved with the OpenMEEG plugin^52,53^ implemented in Brainstorm. We used the weighted minimum norm as an inverse solution method, with Brainstorm’s default parameter settings.^54^

### Parametrizing brain spectra

Prior to performing the parameterization of brain spectral components, source activity was downsampled to 68 cortical regions of interest (ROIs) according to the Desikan–Killiany atlas.^55^ ROI time series were obtained as the mean time series across ROI vertices.^56^ For each ROI time series, the power spectrum was calculated over the continuous signal using Welch’s method (Hann window, length 5 s, overlap 50%). We subsequently parameterized the ROI spectra into periodic and aperiodic components using the ‘fooof’ Python library.^16^ The FOOOF (specparam) algorithm estimates the aperiodic component of the power spectrum before iteratively estimating periodic components, resulting in a parameterized model of the neural power spectrum. Spectra were parameterized in the 1–35 Hz range using default Python hyperparameters; specifically: peak width limits = [0.5, 12], maximum number of peaks = inf, peak threshold = 2, minimum peak height = 0.0, aperiodic mode = ‘fixed’. The ‘fixed’ mode assumes a single 1/f-like characteristic to the aperiodic component, meaning it appears linear across all frequencies in log-log space, while the ‘knee’ mode is used to take into account the presence of bends in the power spectrum distribution in log-log space. In our data, no bends were detected in the log-log space of the PSD, and for this reason, a ‘fixed’ model was the most appropriate. The ‘fixed’ (no knee) model of aperiodic activity results in an aperiodic model of the form: A = B - log(FX) where A is the model of aperiodic activity at sampled frequencies, B is the offset, X is the exponent and F is the set of sampled frequencies.

### Cortical gene expression

Regional microarray expression data were obtained from six post-mortem brains of the AHBA (http://human.brain-map.org/).^57^ We used the abagen toolbox (https://github.com/netneurolab/abagen)^35^ to process and map the data to the 68 ROIs of the Desikan–Killiany parcellation.^55^ To begin, the microarray probes were re-annotated using information provided by Arnatkeviciute and colleagues.^58^ The re-annotated information discarded probes without a reliable match to genes. Probes were then filtered based on their expression intensity relative to background noise.^59^ Specifically, probes with an intensity less than background in ≥50% of samples were discarded. When multiple probes indexed the expression of the same gene, the probe with the most consistent pattern of regional variation (i.e. differential stability);^57^ across donors was selected. Differential stability was calculated as follows:


$$\;\Delta s(p)=\;\frac{1}{\left(\begin{array}{c} N\\ 2 \end{array}\right)}\overset{N-1}{\underset{i=1}{\sum }}\;\overset{N}{\underset{\;j=i+1}{\sum }}\;\rho [B_{i}(p),B_{j}(p)]$$

where *ρ* is Spearman’s rank correlation of the expression of a single probe *P* across regions in two donor brains B*_i_* and B*_j_* and *N* is the total number of donors. This procedure retained 15 656 probes, each representing a unique gene.

To reduce the potential for misassignment, sample-to-region matching was constrained by hemisphere and cortical/subcortical divisions. Samples were assigned to the closest region in the parcellation. Additionally, a threshold distance of 2 mm from the original sample location to the parcellation is applied to avoid inaccurate mapping of samples that are located too far from the region.^58^ All tissue samples not assigned to a brain region in the provided atlas were discarded.

Inter-subject variation was addressed by normalizing tissue sample expression values across genes using a robust sigmoid function^60^


$$x_{\mathrm{norm}}=\;\frac{1}{1+\exp\left(-\;\frac{\left(x-\left\langle x\right\rangle \right)}{\mathrm{IQ}R_{x}}\right)}$$

where ⟨x⟩ is the median and IQR*_x_* is the normalized interquartile range of the expression of a single tissue sample across genes. Normalized expression values were then rescaled to the unit interval.


$$x_{\mathrm{scaled}}=\;\frac{x_{\mathrm{norm}}-\;\min(x_{\mathrm{norm}})}{\max(x_{\mathrm{norm}})\;-\;\min(x_{\mathrm{norm}})}$$

Gene expression values were then normalized separately for each donor across regions using an identical procedure. Scaled expression profiles were finally averaged across donors, resulting in a single matrix with rows corresponding to brain regions and columns corresponding to the retained 15 656 genes.

Based on a recent panel of epilepsy-related genes,^61^ we extracted a set of 22 genes linked with the regulation of intra and intercellular E/I balance. These selected genes regulate the functioning of sodium-potassium channels, GABA and *N*-methyl-D-aspartate (NMDA) receptors, seizure susceptibility and the presence of focal cortical dysplasia. A detailed list of the included genes and the associated functions is provided in Supplementary Materials (see Supplementary Table 1). Importantly, the set of 22 genes was selected prior to conducting the statistical analyses.

### Statistical analysis

**Figure 1:**
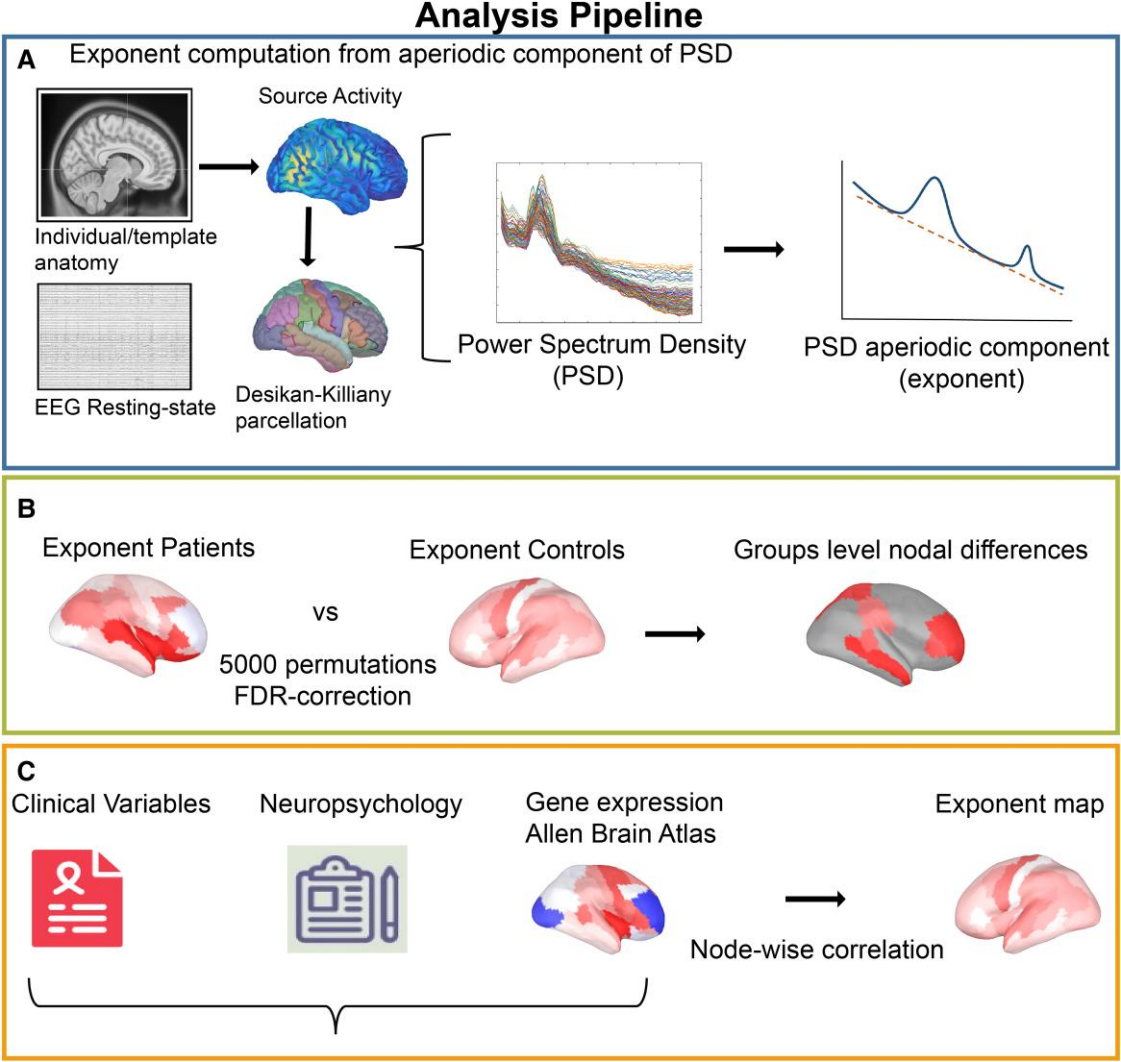
**Analytical pipeline.** The present figure displays the analytical steps performed. Panel **A** follows the steps for extracting aperiodic exponents from resting-state EEG recordings. Panel **B** graphically explains the statistical comparison performed. Panel **C** represents the correlational analysis between clinical variables, neuropsychological assessment, gene expression and aperiodic exponent.

We first contrasted the exponent value of all patients with epilepsy against controls for each of the ROIs, using a mass univariate non-parametric permutation approach (5000 permutations).^62^ This method consists of performing a statistical test, in our specific case an independent sample *t*-test, for every variable of interest (the exponent value for each ROI). Successively, the between-group condition assignments (i.e. TLE patients or controls) are iteratively permuted and the test is performed a sufficient number of times (5000) to have an empirical null distribution of the test statistic under the null hypothesis of no difference between the two groups.^63^ The null distribution obtained by the permutation is used to derive the probability of the observed difference (two-tails) and thus perform the statistical inference. We then repeated the same analysis contrasting specific patient groups against healthy controls, namely left-TLE, right-TLE and bilateral-TLE. A similar analysis was performed for the offset value. Additionally, in order to assess the frequency of E/I alteration across subjects, we calculated the within-subject distribution of the exponent across parcels (analysis details and results are reported in Supplementary Materials, see Supplementary Fig. 1). Successively, we performed Spearman’s correlation between the exponent and clinical features (age of onset, epilepsy duration and number of ASMs) as well as neuropsychological test scores. Additionally, to better disentangle the relationship across E/I balance, cognition and clinical variables, we performed multiple linear regression. The model details are reported in Supplementary Materials. Finally, correlation analysis was also computed between aperiodic exponent values and gene expression. Specifically, we correlated, across ROIs, the gene expression level (separately for each gene of interest) and the relative aperiodic exponent value. Notably, exponent-gene correlation was performed in both TLE patients and controls, to highlight specific patterns related to epilepsy. The significance level was set to *P* < 0.05. The alpha inflation due to multiple comparisons was controlled with the false discovery rate (FDR) procedure,^64^ across ROIs (*N* = 68) or genes (*N* = 22), as appropriate. Brain plots were obtained using the enigma-toolbox^65^ (see Fig. 1 for a graphical representation of the pipeline).

## Results

### Exponent differences in TLE versus controls

**Figure 2:**
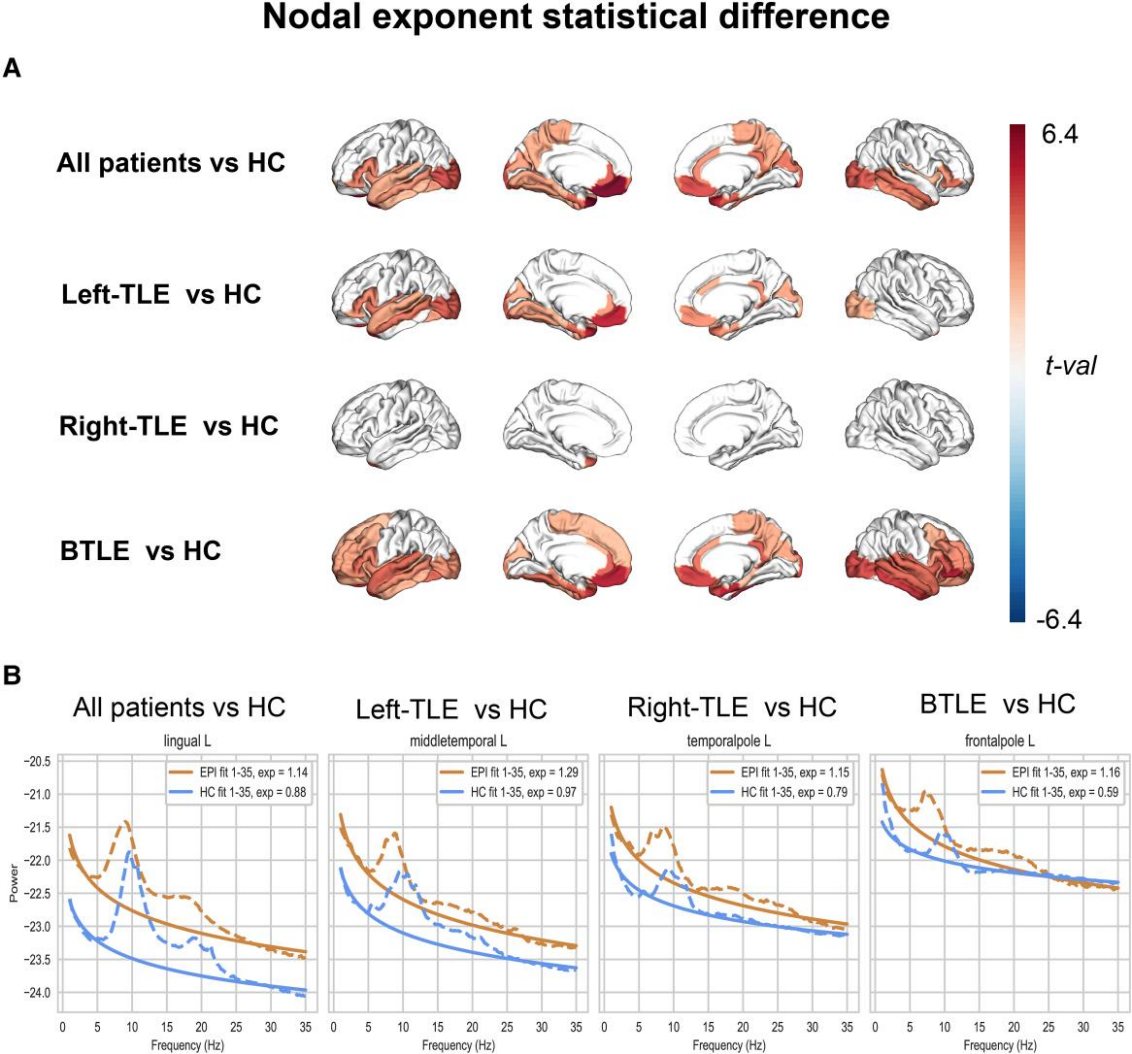
**Statistical difference across groups of the nodal aperiodic exponent.** Panel **A** displays the statistical difference of the aperiodic exponent comparing all patients with temporal lobe epilepsy (All patients; *N* = 67), left temporal lobe epilepsy (left-TLE; *N* = 30), right temporal lobe epilepsy (right-TLE; *N* = 17) and BTLE (*N* = 20) versus HC (*N* = 35). Panel **B** shows the PSD and the fit of the aperiodic component (fit) and the corresponding exponent values (exp) of the regions with the maximum *t*-value in each of the comparisons performed for patients with epilepsy (orange line) and HC (blue line). Specifically, we reported lingual left (lingual L), middle temporal left (middle temporal L), temporal pole left (temporal pole L) and frontal pole left (frontal pole L), derived from the Desikan–Killiany atlas. The statistical test used was permutation two-tail *t*-test. Cortical maps are thresholded at *P* < 0.05 after FDR correction.

We observed that patients with TLE are characterized by a larger aperiodic exponent (i.e. steeper spectral slope, lower E/I) as compared to healthy controls. Significant differences were mainly observed in regions involved in seizure propagation such as superior and inferior temporal gyri, entorhinal cortex and temporal pole, anterior cingulate cortex and in the posterior quadrant (cuneus and visual cortex) (*t*_max_ = 6.248; *p*_adjusted_ < 0.001; see Fig. 2A). When considering the three groups separately, patients with both right- and left-TLE showed a larger exponent in the left temporal areas (*t*_max_ = 5.136; *p*_adjusted_ = 0.0001), whereas bitemporal epilepsy (BTLE) showed an increase in both temporal and frontal regions, bilaterally (*t*_max_ = 5.247; *p*_adjusted_ = 0.0002) (see Fig. 2). All regional statistical values (*t-*value and the corresponding *P-*value for each significant ROI) are provided in Supplementary Materials (Supplementary Tables 2–5). Similar results regarding the involvement of temporal areas were also detected when considering the offset. The biological meaning of the offset is less known as compared to the exponent with respect to the E/I balance,^18^ and for this reason, we focused on the interpretation of the aperiodic exponent. We report the offset results in Supplementary Materials (see Supplementary Fig. 2).

### Clinical variable and neuropsychology correlations

**Figure 3:**
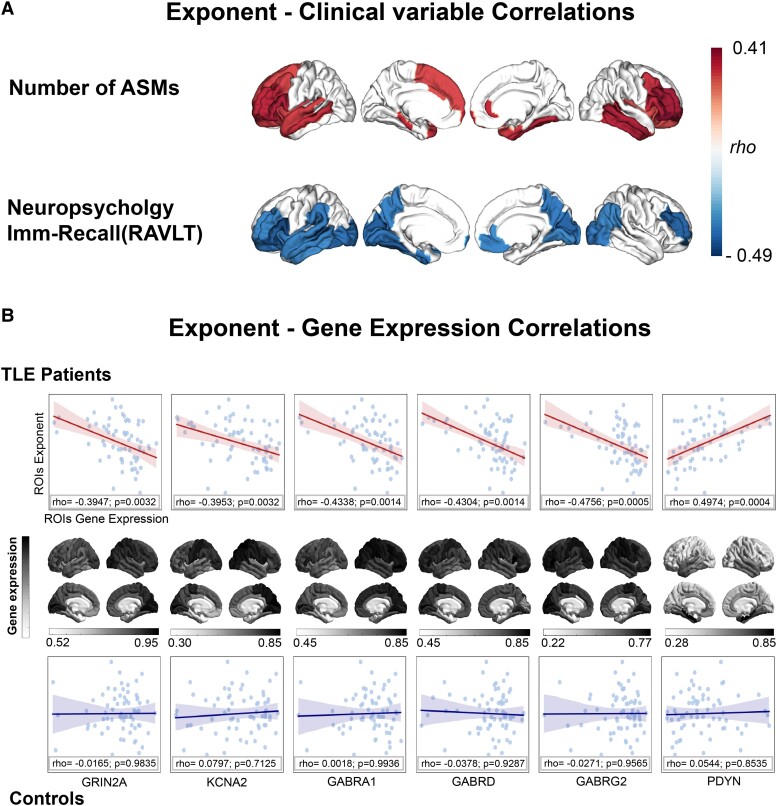
**Exponent correlation with clinical and transcriptomics variables.** Panel **A** shows the spatial distribution on the cortex of the node-wise correlation (Spearman’s rho) between the region aperiodic exponent and the number of ASMs and the short-term verbal memory functioning measured by the Imm-Recall of the RAVLT. Panel **B** displays the exponent-gene expression correlation both for patients with TLE patients (upper line) and the control group (controls; lower line). In the correlation plots, each dot represents the related gene expression level in the *x*-axis and the relative aperiodic exponent level in the *y*-axis of all the ROIs of the atlas. The shaded area around the correlation line represents the 95% confidence interval. The brain plots in black and white illustrate the node-wise cortical expression of the corresponding gene.

A significant positive relationship between the aperiodic exponent and the number of ASMs was found in the entire group of patients, suggesting that a larger number of antiseizure medications corresponded to lower E/I. The correlation was spatially expressed in the medial temporal regions as well as in the frontal gyrus and dorsolateral prefrontal cortex (DLPFC) (*ρ*_max_ = 0.41; *p*_adjusted_ = 0.026; see Fig. 3A). A significant negative correlation was found between aperiodic exponent and short-term verbal memory (RAVLT) in the left temporal regions, the DLPFC, and cuneus bilaterally, with larger exponent (lower E/I) linked to a lower cognitive performance (*ρ*_max_ = −0.49; *p*_adjusted_ = 0.021; see Fig. 3A). All regional statistical values (Spearman’s *ρ* and the corresponding *P*-value for each significant ROI) are provided in Supplementary Materials (Supplementary Tables 6 and 7). In line with the correlation results, the multiple regression model identified a main effect on the aperiodic exponent of the number of ASMs (*F* = 9.423; *P* = 0.004) and the memory score (*F* = 8.668; *P* = 0.005). Importantly, no interaction effect between ASMs and memory score was observed (*F* = 0.9204; *P* = 0.343) (see Supplementary Fig. 3). Second, while explaining the memory score, we observed a significant effect of the E/I value (*F* = 8.079; *P* = 0.007), but no main effect of the number of ASMs (*F* = 0.087; *P* = 0.785) or the interaction between ASMs and E/I value (*F* = 0.227; *P* = 0.636). Note that the analysis of cognition was limited to the RAVLT-immediate recall (Imm-Recall) variable, which was the only significant neuropsychological test in the correlation analysis.

### Exponent-gene correlations results

The correlation between aperiodic exponent and cortical gene expression displayed a specific pattern differentiating TLE patients and controls. Specifically, we observed a higher cortical expression of the genes related to the functioning of sodium channels (*KCNA2*; *ρ* = −0.395; *p*_adjusted_ = 0.0032) and GABA receptors (*GRIN2A*; *ρ* = −0.394; *p*_adjusted_ = 0.0032; *GABRA1*; *ρ* = −0.433; *p*_adjusted_ = 0.0014; *GABRD*; *ρ* = −0.403; *p*_adjusted_ = 0.0014; *GABRG2*; *ρ* = − 0.475; *p*_adjusted_ = 0.0005) were associated with a lower exponent value. Additionally, we observed an opposite pattern in a gene involved in the self-regulation of the seizure through the peptide ligands of opioid receptors (PDYN; *ρ* = 0.497; *p*_adjusted_ = 0.0004). By contrast, we observed no significant correlation in the control group (see Fig. 3B).

## Discussion

We leveraged the exponent of the aperiodic component of the power spectrum from source-derived hdEEG signals as a non-invasive and cost-efficient proxy of excitation/inhibition alterations in patients with epilepsy. We observed that patients with TLE were characterized by larger aperiodic exponent values compared to healthy controls. The statistical difference was maximally expressed in temporal regions, as well as dorsal frontal areas, the cingulate cortex and the precuneus (Fig. 2A). This E/I imbalance displayed sensitivity to the lateralization of the focus with a left dominant exponent increase in left-TLE and a bilateral distribution in BTLE. Converging evidence suggests that higher values of the aperiodic exponent are indicators of system E/I balance shifted toward inhibition.^16,17^ To this end, our results suggest that patients with TLE display a more predominant inhibitory component, as assessed from non-invasive and perturbation-free methods. Our cohort includes medically refractory epilepsy patients treated with antiseizure medications. A significant link between the number of medications and E/I shift toward inhibition was indeed observed both from correlation and multiple regression analysis. Importantly, the spatial configuration of the relationship suggests that fronto-temporal areas were the most affected. Multiple interpretations can account for the exponent-pharmacology relationship. First, converging evidence from neuroimaging studies has shown that medial prefrontal and temporoparietal cortices display longer intrinsic neuronal time scales (INT).^66,67^ INTs can be interpreted as the degree to which spontaneous brain activity correlates with itself over time, setting the duration over which brain regions integrate inputs.^68^ Patients with TLE are characterized by longer INTs in the temporal areas.^69^ Relevantly, recent findings suggest that INTs have a direct link with the decay of the PSD and therefore the exponent value.^70^ The regions characterized by slower intrinsic timescales could be the most sensitive to the ASMs, resulting in an increase of low-frequency power and a larger exponent. Second, homeostatic plasticity mechanisms would explain this spatial distribution. Homeostatic plasticity enables the tuning of synaptic activity in relation to environmental stimuli, in which the magnitude and direction of synaptic plasticity are adjusted according to the recent history of postsynaptic activity.^71,72^ Regions belonging to the epileptic network exhibit altered E/I balance,^73^ potentially resulting in a higher sensitivity to the effect of antiseizure medications.^74^ Therefore, antiseizure medication may contribute to a neuroplastic shift in E/I balance in these regions, which could represent the effect we are capturing in the ASMs-aperiodic exponent correlation.^75^ This interpretation would be consistent with previous transcranial magnetic stimulation studies, which have detected homeostatic plasticity effects in the TLE.^72,76,77^ Dysregulation of E/I balance has been proposed as one of the neurophysiological mechanisms explaining performance reduction of cognitive functioning^78^ and may represent the mechanism explaining the relationship between the exponent and memory function observed in the correlation and multiple regression model. Importantly, the multiple regression model did not provide a direct link between the number of ASMs and memory outcome. The exponent-memory relationship displayed a spatial configuration involving the left temporal and bilateral dorsolateral prefrontal cortices. Neuroimaging studies identified left temporal areas as the scaffolding regions of verbal memory.^79-81^ However, the information retention and recall engage a distributed network also involving fronto-parietal regions.^82-85^ In light of this, our findings suggest that altered E/I balance in the distributed network underlying memory may be one of the neurophysiological mechanisms explaining impairments of this cognitive domain characterizing patients with TLE.^86-88^ The response properties of neurons are indeed shaped by the balance between co-activated inhibitory and excitatory synaptic inputs.^89^ Regulation of E/I balance is a multifactorial process related to neuro-biophysical properties and dynamics mediated by neurotransmission and channel activity.^90,91^ Computational and biophysical models show that GABA and NMDA receptors as well as sodium-potassium channels are the main regulators of E/I at multiple scales, from single cell to vast neuronal assemblies.^17,92-95^ By integrating source EEG functional and transcriptional atlas data, we tested the hypothesis that gene expression could covary with E/I, providing information of the potential mechanisms underlying E/I deregulation in TLE. We observed a correlation of the non-invasive measure of E/I balance with the expression of genes regulating GABA receptors and potassium channels exclusively in the patients’ group. Specifically, increased cortical expression of *GABRA1*, *GRIN2A*, *GABRD* and *GABRG2* are associated with lower values in the aperiodic exponent. This correlational pattern is also observed in the gene regulating potassium voltage-gated channel subfamily A member 2, namely *KCNA2*. Noticeably, alterations of genes’ expression regulating GABA receptor subunits, as well as potassium channels, have been linked with the disruption of inhibitory network development and/or activity modulation, being considered as one of pathophysiological mechanisms underlying multiple types of epilepsy.^96-98^ Additionally, we also observed a relationship between the aperiodic exponent and the cortical expression of the *PDYN* in the TLE patients group. This gene is involved in the synaptic plasticity via kappa-opioid receptors^99,100^ and encodes for the anticonvulsant peptide dynorphin, a strong candidate for a seizure suppressor gene and thus a possible modulator of susceptibility to TLE.^101^ Notably, the correlation patterns between aperiodic exponent and gene expression were exclusively identified in the clinical group. Our findings suggest that the genes with lower expression in the fronto-temporal regions are negatively correlated with the exponent values. Assuming the larger aperiodic exponent value as an expression of the E/I balance dysregulation, we may hypothesize that an increase in gene expression, especially in the temporal areas, would promote the homeostatic process of E/I regulation. A second hypothesis could propose the vulnerability to epilepsy in mutations specifically located in fronto-temporal regions of the investigated gene. Relevantly, literature has identified gain or a loss of function of *KCN2A*, *GRIN2A* as well as *GABRD* in relation to epilepsy.^102-105^ Using normative transcriptomics atlas data to contextualize functional alterations in epilepsy has intrinsic limitations from a mechanistic perspective. In fact, public genetic atlases are derived from an independent sample of individuals. However, these data represent a methodological unicum of their kind, being a useful tool to understand the link between micro to macro-scale levels of network function and dysfunction, and has proven useful in recent studies of several neurological disorders other than epilepsy.^106-108^

## Limitations

We cannot rule out that the exponent of the PSD captures other signal properties beyond E/I balance (e.g. criticality^109^). The largest part of the literature investigating the PSD features in epilepsy has seldom disentangled the periodic from the aperiodic component. Patients with TLE are known to have increased slow activity.^110,111^ The mechanisms sustaining these phenomena are many-fold and involve structural damage,^112^ cortical plasticity^113,114^ and intracranial epileptiform abnormalities which appear as slow activity on the surface due to the conductance properties of skin and skull.^111,115,116^ Simultaneous invasive and non-invasive recordings may be beneficial to parametrize the influence of these components on the exponent-derived estimation of E/I.^11^ Additionally, the present results have limitations in the identification of lateralization and it would be overly simplistic to attribute direct clinical translation to our results. In fact, literature supports that a convergence of multimodal investigation is necessary for an accurate identification of lateralization.^117^ Moreover, recent findings suggest that patients with TLE may experience seizures occurring in either hemisphere over long periods of time, so the most promising approach is chronic recordings, potentially with invasive tools.^118^ However, we provide relevant evidence that this index may represent a useful marker. Future studies with longitudinal assessment of the scalp EEG-derived aperiodic exponent might be contributory in delineating clinical applications of this measure.

## Conclusions

Our results reinforce the aperiodic exponent as a functional measure of E/I of the intrinsic functional organization on a large-scale network in patients with TLE, displaying sensitivity to the lateralization of the epileptogenic hemisphere. Our findings support the functional relevance of the E/I assessed based on the aperiodic exponent for cognition, with a dependance on antiseizure medication. Syndrome-specific functional-transcriptomic signatures could provide information on the altered mechanisms underlying E/I balance dysregulation implied in the etiopathogenesis of epilepsy. Transcriptomics data may also provide a potential link between E/I balance regulation and cognitive impairment in TLE. Dysregulation in the expression of the investigated genes in fronto-temporal areas may lead to an altered balance of E/I in these regions. Therefore, dysregulation of E/I in the fronto-temporal network, which scaffolds high-level cognitive functions, offers a potential mechanism underlying memory impairment characterizing patients with TLE. Finally, in this study we evidenced the potential usage of a cost-efficient and non-invasive measure of the E/I balance. Further studies may highlight the potential value of integrating the simple and informative measures presented here in the assessment of TLE patients, including applications in long monitoring, to investigate circadian changes of E/I in relation to ictal manifestation.

## Supplementary Material

fcae231_Supplementary_Data

## Data Availability

The data that support the findings of this study are available on request to the corresponding author. The raw data are not publicly available due to privacy or ethical restrictions. All the scripts are available at the following GitHub page: https://github.com/simone-cuozzo/FOOOF_epilepsy.
