# Supplementary material for: Excitation/Inhibition balance relates to cognitive function and gene expression in temporal lobe epilepsy: a high density EEG assessment with aperiodic exponent
Source: Brain Commun. 2024 Jul 8;6(4):fcae231. doi: 10.1093/braincomms/fcae231 (PMC11272395; doi:10.1093/braincomms/fcae231)
Supplement: fcae231_Supplementary_Data [file fcae231_supplementary_data.docx]

**Supplementary Materials**

**Frequency of E/I alteration across subject.**


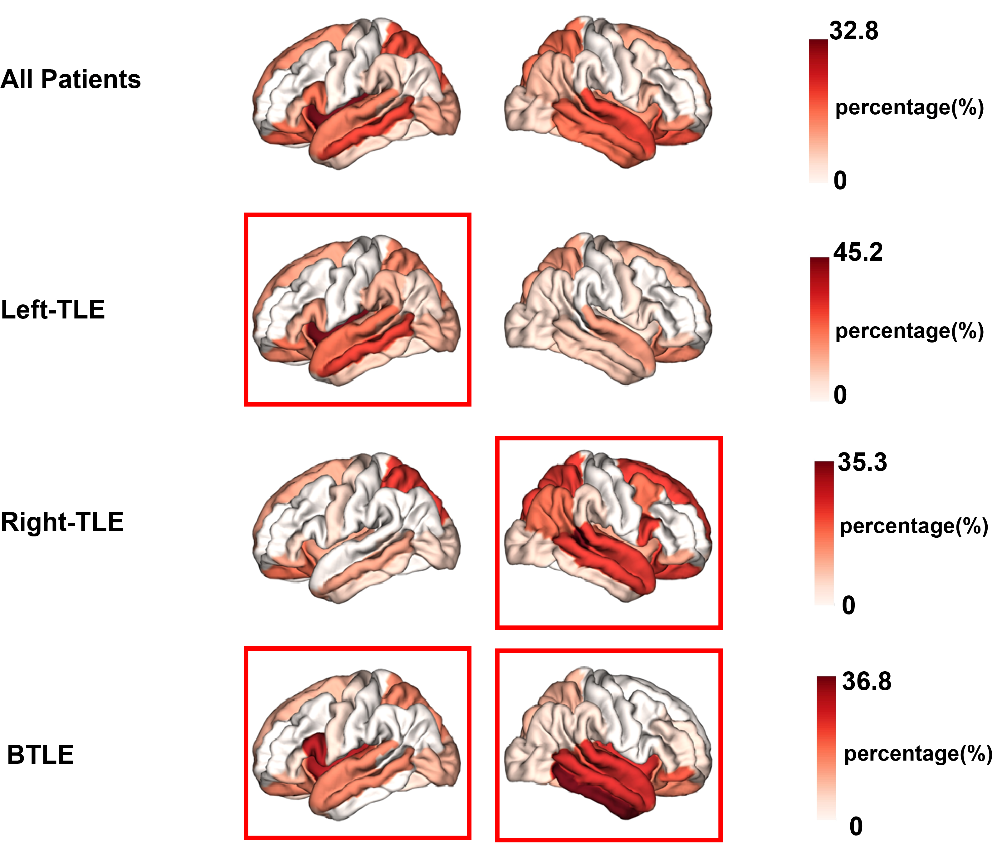
In order to assess the frequency of E/I alteration across subjects, we calculated the within subject distribution of the exponent across parcels. Next, we applied a threshold to the brain maps so to retain only values above the 90^th^ percentile. Finally, we generated maps with each parcel showing the frequency of subjects with an exponent above threshold for that location. As showed in the Supplementary Fig.1F patients with Left temporal lobe epilepsy more often had increased exponent in the left temporal areas, whereas patients with right temporal lobe epilepsy had an opposite behavior. Bilateral patients showed more often an involvement of both temporal areas. While these findings are qualitative, they provide evidence that the value of E/I are sensitive to the lateralization.

**Supplementary Figure 1: Frequency of altered E/I across subjects.** we calculated the within subject distribution of the exponent across parcels. We then applied a threshold to the brain maps so to retain only values above the 90^th^ percentile. The present image shows for each parcel the frequency of subjects with an exponent above threshold for that location.

**
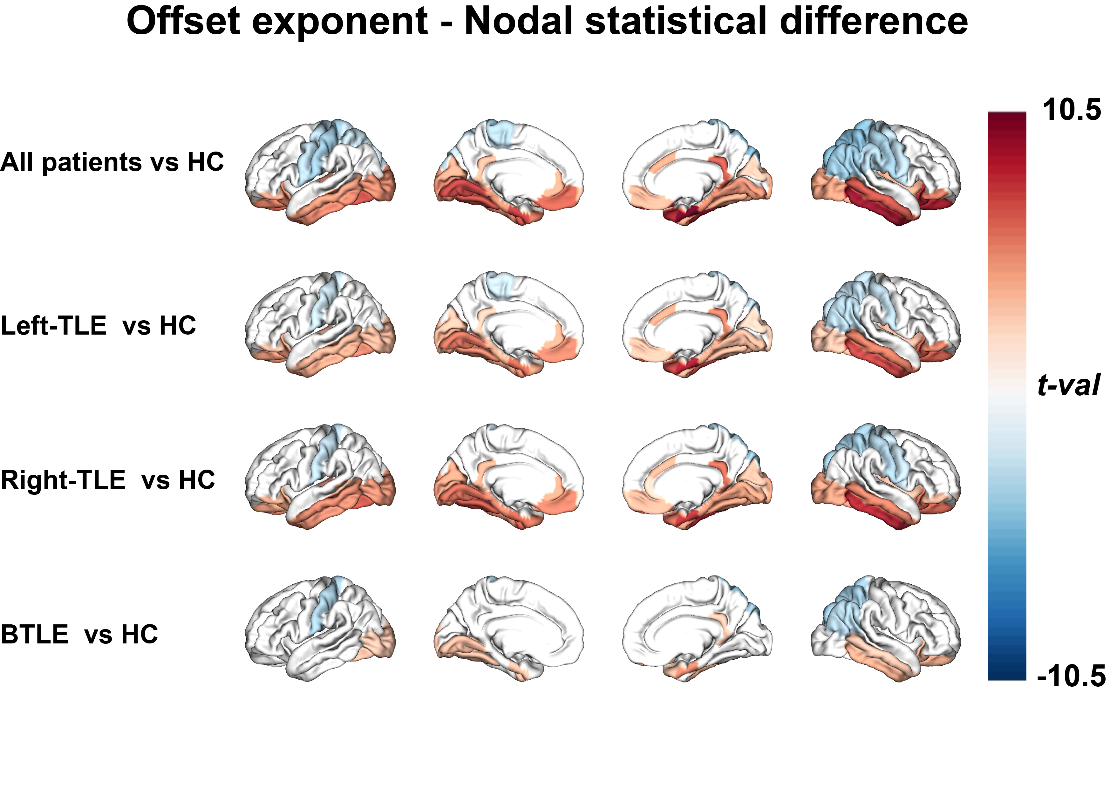
**

**Supplementary Figure 2. Statistical difference across groups of the nodal offset value.** The figure displays the statistical difference of the offset value comparing all patients with temporal lobe epilepsy (TLE; N=67), left temporal lobe epilepsy (Left-TLE; N=30), right temporal lobe epilepsy (Right-TLE; N=17) and bilateral temporal lobe epilepsy (BTLE; N=20) vs. healthy controls (HC; N=35). All the results are corrected with False Discovery rate approach.

**Multiple regression analysis**

In order to disentangle the relationship between E/I balance, number of antiseizure medications (ASMs) and memory functioning, we performed multiple regression approach. We used the average value of the exponent per subject as dependent variable and the following predictors: Age of epilepsy onset, patient’s age, memory function (RAVLT - immediate recall), Number of Drugs, Epilepsy duration, Sex. In the model we also included the interaction between Number of Drugs and memory score. To test in more details, the effect of ASM on reduced cognitive performance, we ran an additional regression model with memory functioning (RAVLT-immediate recall) as dependent variable and the following factors: Number of ASMs, Average value of the aperiodic exponent (E/I) per subjects, age of onset, epilepsy duration and gender. We also tested the interaction between the Number of ASMs and the E/I value. The analysis was performed with R open tool, in the following way

Model 1<- *lm (E/I~ Age of epilepsy onset + patient’s age + memory function*Number of Drugs + Epilepsy duration + Sex)*

Model 2<- lm (Memory *~ Age of epilepsy onset + patient’s age + E/I*Number of Drugs + Epilepsy duration + Sex)*

This analysis of Model 1 revealed two main effects:

Number of Drugs: F = 9.423; p-val = 004. The main effect plot shows an increase of the average aperiodic exponent in relation to the increase of the number of ASMs.

Memory functioning (RAVLT-immediate recall): F=8.668; p-val = 005. This resulted in a reduced memory function related to larger exponent values (increased inhibition).

No significant interaction between ASM and memory functioning was detected: F = 0.9204; p-val = .343.


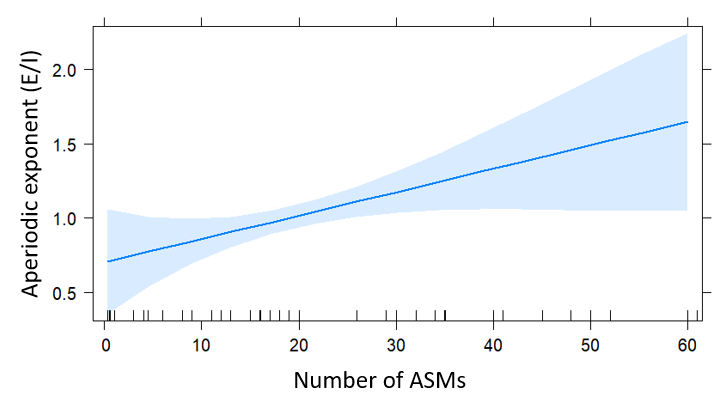

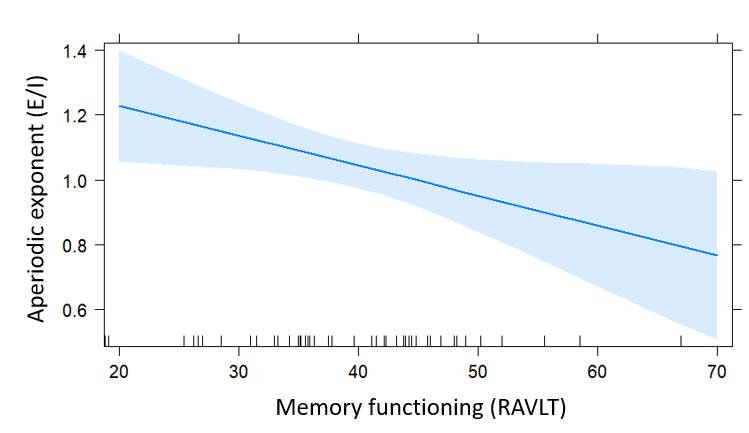


**Supplementary Figure 3.** Main effect in the model 1 of Number of antiseizure medications and memory functioning of the aperiodic exponent.

This analysis of Model 2 revealed one main effects:

E/I value on memory functioning: F=8.079; p-val = .007, but no main effect of the number of ASMs (F=0.087; p-val = .785) or the interaction between ASMs and E/I value (F=0.227; p-val = .636). This suggests that ASMs are a coincident factor in our results but they are not the element entirely driving our findings

| **Studied Genes** | **Area of Interest** |
| --- | --- |
| LGI1 | Temporal Epilepsy |
| CNTNAP2 | Cortical dysplasia |
| GRIN2A | Focal epilepsy |
| SCN1A | Sodium channels |
| SCN1B | Sodium channels |
| SCN2A | Sodium channels |
| KCNA2 | Potassium channels |
| KCNB1 | Potassium channels |
| KCNC1 | Potassium channels |
| KCNMA1 | Potassium channels |
| KCNQ3 | Potassium channels |
| KCNT1 | Potassium channels |
| GABRA1 | Gaba receptor |
| GABRB1 | Gaba receptor |
| GABRB2 | Gaba receptor |
| GABRB3 | Gaba receptor |
| GABRD | Gaba receptor |
| GABRG2 | Gaba receptor |
| GRIN2B | NMDA receptor |
| GRIN2D | NMDA receptor |
| GRINA | NMDA receptor |
| PDYN | Seizure suppression |

**Supplementary Table 1.** The present table enlists the gene and the corresponding function, selected for the gene expression-exponent correlation.

| **ALL PATIENTS vs. CONTROLS** | | | |
| --- | --- | --- | --- |
| **Regions** | **t-values** | **p-values** | **effect size (Cohen’s D)** |
| bankssts R | 2,7287 | 0,0176 | 0.5299 |
| caudalanteriorcingulate R | 3,1853 | 0,0064 | 0.5892 |
| cuneus L | 2,9242 | 0,0119 | 0.5678 |
| cuneus R | 3,6811 | 0,0021 | 0.6989 |
| entorhinal L | 3,9091 | 0,0014 | 0.7481 |
| entorhinal R | 4,2011 | 0,0007 | 0.8505 |
| frontalpole L | 3,4686 | 0,0036 | 0.6077 |
| frontalpole R | 3,3505 | 0,0043 | 0.5821 |
| fusiform L | 2,9655 | 0,0119 | 0.6889 |
| inferiortemporal L | 2,3066 | 0,0448 | 0.4974 |
| inferiortemporal R | 3,2686 | 0,0056 | 0.6699 |
| insula L | 3,0884 | 0,0083 | 0.6148 |
| insula R | 2,491 | 0,0283 | 0.5070 |
| isthmuscingulate R | 3,7996 | 0,0017 | 0.7129 |
| lateraloccipital L | 4,3179 | 0,0007 | 0.8795 |
| lateraloccipital R | 4,1457 | 0,0007 | 0.8090 |
| lingual L | 2,8372 | 0,0138 | 0.5763 |
| medialorbitofrontal L | 6,2482 | 5.5e-07 | 1.2105 |
| medialorbitofrontal R | 4,3958 | 0,0005 | 0.8455 |
| middletemporal L | 3,0668 | 0,0089 | 0.6492 |
| middletemporal R | 3,7958 | 0,0017 | 0.7178 |
| paracentral L | 2,8148 | 0,0138 | 0.4946 |
| paracentral R | 2,85 | 0,0134 | 0.5010 |
| parahippocampal L | 2,6845 | 0,0196 | 0.5937 |
| parsopercularis L | 3,3897 | 0,0042 | 0.6495 |
| parsorbitalis L | 3,2909 | 0,005 | 0.6039 |
| parsorbitalis R | 3,7517 | 0,0017 | 0.7003 |
| parstriangularis L | 3,4789 | 0,0036 | 0.6713 |
| parstriangularis R | 3,0788 | 0,0083 | 0.5971 |
| precuneus L | 2,6441 | 0,0196 | 0.4714 |
| precuneus R | 2,6955 | 0,0181 | 0.4818 |
| rostralanteriorcingulate L | 4,1638 | 0,0007 | 0.7870 |
| rostralanteriorcingulate R | 3,4396 | 0,0037 | 0.6238 |
| superiortemporal L | 2,5153 | 0,028 | 0.5397 |
| temporalpole L | 6,3984 | 5.5e-07 | 1.2665 |
| temporalpole R | 5,0209 | 5.4e-05 | 0.9201 |
| transversetemporal L | 2,5597 | 0,025 | 0.5170 |

**Supplementary Table 2.** The present table enlists the *t*- and the FDR corrected *p-values*, as well as the effect size, for each significant region in the comparison of the aperiodic exponent between the whole group of patients with epilepsy and healthy controls

| **LEFT TLE vs. CONTROLS** | | | |
| --- | --- | --- | --- |
| **Regions** | **t-values** | **p-values** | **effect size (Cohen’s D)** |
| bankssts L | 2,6589 | 0,0348 | 0.6469 |
| caudalanteriorcingulate R | 2,649 | 0,0348 | 0.6697 |
| cuneus L | 2,6527 | 0,0348 | 0.6606 |
| cuneus R | 2,8388 | 0,0288 | 0.7134 |
| entorhinal L | 3,9742 | 0,0033 | 0.9909 |
| entorhinal R | 3,0777 | 0,0167 | 0.7613 |
| fusiform L | 2,8529 | 0,0288 | 0.6865 |
| insula L | 3,6415 | 0,0062 | 0.9020 |
| isthmuscingulate R | 3,3049 | 0,0118 | 0.8287 |
| lateraloccipital L | 4,0948 | 0,0027 | 1.0061 |
| lateraloccipital R | 2,5161 | 0,0437 | 0.6311 |
| lingual L | 2,7197 | 0,0337 | 0.6692 |
| medialorbitofrontal L | 5,1364 | 0,0001 | 1.2744 |
| medialorbitofrontal R | 3,2228 | 0,0131 | 0.8073 |
| middletemporal L | 3,5648 | 0,0062 | 0.8688 |
| parahippocampal L | 2,5468 | 0,0411 | 0.6203 |
| parsopercularis L | 3,2769 | 0,0118 | 0.8141 |
| parsorbitalis L | 2,7445 | 0,0337 | 0.6921 |
| parstriangularis L | 3,5696 | 0,0062 | 0.8912 |
| rostralanteriorcingulate L | 3,0908 | 0,0167 | 0.7794 |
| superiortemporal L | 2,6893 | 0,0345 | 0.6541 |
| temporalpole L | 5,2419 | 0,0001 | 1.3047 |
| temporalpole R | 3,5982 | 0,0062 | 0.9074 |

**Supplementary Table 3.** The present table enlists the *t*- and the FDR corrected *p-values,* as well as the effect size, for each significant region in the comparison of the aperiodic exponent between the group of patients with left temporal lobe epilepsy (Left-TLE) and healthy controls

| **RIGHT TLE** | | | |
| --- | --- | --- | --- |
| **Regions** | **t-values** | **p-values** | **effect size (Cohen’s D)** |
| temporalpole L | 3,9774 | 0,0224 | 1.1294 |

**Supplementary Table 4.** The present table enlists the *t*- and the FDR corrected *p-values,* as well as the effect size, for each significant region in the comparison of the aperiodic exponent between the group of patients with right temporal lobe epilepsy (Right-TLE) and healthy controls

| **BILATERAL TLE** | | | |
| --- | --- | --- | --- |
| **Regions** | **t-values** | **p-values** | **effect size (Cohen’s D)** |
| bankssts L | 2,5844 | 0,0236 | 0.7389 |
| bankssts R | 4,1348 | 0,0013 | 1.2414 |
| caudalanteriorcingulate L | 2,5594 | 0,0271 | 0.8184 |
| caudalanteriorcingulate R | 2,9396 | 0,0132 | 0.9359 |
| caudalmiddlefrontal L | 2,4715 | 0,0303 | 0.8105 |
| caudalmiddlefrontal R | 2,4733 | 0,0298 | 0.7790 |
| cuneus L | 2,4796 | 0,0298 | 0.7888 |
| cuneus R | 2,8236 | 0,0173 | 0.9155 |
| entorhinal L | 4,1744 | 0,0013 | 1.2742 |
| entorhinal R | 5,2479 | 0,0002 | 1.4768 |
| frontalpole L | 4,0355 | 0,0017 | 1.3432 |
| frontalpole R | 4,0217 | 0,0017 | 1.3099 |
| fusiform L | 3,8975 | 0,0013 | 0.9936 |
| inferiortemporal L | 2,4883 | 0,0277 | 0.7137 |
| inferiortemporal R | 4,7822 | 0,0005 | 1.3613 |
| insula L | 3,1311 | 0,0081 | 0.9194 |
| insula R | 4,0093 | 0,0013 | 1.1162 |
| isthmuscingulate R | 4,1153 | 0,0013 | 1.2454 |
| lateraloccipital L | 3,1139 | 0,0089 | 0.9785 |
| lateraloccipital R | 4,38 | 0,0012 | 1.3163 |
| lateralorbitofrontal L | 2,6286 | 0,0235 | 0.8133 |
| lateralorbitofrontal R | 3,0227 | 0,0111 | 0.9611 |
| medialorbitofrontal L | 5,3315 | 0,0003 | 1.7007 |
| medialorbitofrontal R | 4,565 | 0,001 | 1.4175 |
| middletemporal L | 3,2124 | 0,0075 | 0.9207 |
| middletemporal R | 3,904 | 0,0019 | 1.2542 |
| paracentral L | 2,5725 | 0,0271 | 0.8663 |
| paracentral R | 2,7004 | 0,0229 | 0.9035 |
| parahippocampal L | 3,4528 | 0,0037 | 0.9282 |
| parahippocampal R | 2,4161 | 0,033 | 0.7760 |
| parsopercularis L | 3,3579 | 0,0068 | 1.0874 |
| parsopercularis R | 3,0794 | 0,0082 | 0.8523 |
| parsorbitalis L | 3,2172 | 0,0081 | 1.0606 |
| parsorbitalis R | 5,4299 | 0,0002 | 1.637 |
| parstriangularis L | 3,8384 | 0,0019 | 1.1635 |
| parstriangularis R | 4,2053 | 0,0013 | 1.2341 |
| precuneus R | 2,6622 | 0,0235 | 0.8862 |
| rostralanteriorcingulate L | 4,0842 | 0,0014 | 1.2870 |
| rostralanteriorcingulate R | 3,7876 | 0,0027 | 1.2528 |
| rostralmiddlefrontal L | 2,8046 | 0,0173 | 0.8919 |
| rostralmiddlefrontal R | 3,281 | 0,0076 | 1.0598 |
| superiorfrontal L | 2,2703 | 0,0451 | 0.7415 |
| superiortemporal L | 3,9273 | 0,0013 | 1.0503 |
| superiortemporal R | 3,8424 | 0,0017 | 1.1087 |
| temporalpole L | 4,809 | 0,0006 | 1.5106 |
| temporalpole R | 4,4478 | 0,0013 | 1.4687 |
| transversetemporal L | 3,1626 | 0,008 | 0.9329 |
| transversetemporal R | 3,1949 | 0,008 | 0.9676 |

**Supplementary Table 5.** The present table enlists the *t*- and the FDR corrected *p-values,* as well as the effect size, for each significant region in the comparison of the aperiodic exponent between the group of patients with bilateral temporal lobe epilepsy (BTLE) and healthy controls

| **Exponent – number of ASMs Spearman Correlation** | | |
| --- | --- | --- |
| **Regions** | **rho** | **p-values** |
| bankssts R | 0,353 | 0,0258 |
| caudalanteriorcingulate L | 0,2839 | 0,0467 |
| caudalmiddlefrontal L | 0,295 | 0,0418 |
| caudalmiddlefrontal R | 0,3146 | 0,0329 |
| entorhinal R | 0,3139 | 0,0329 |
| frontalpole L | 0,3529 | 0,0258 |
| frontalpole R | 0,3432 | 0,027 |
| fusiform R | 0,3359 | 0,027 |
| inferiortemporal R | 0,3444 | 0,027 |
| insula L | 0,2992 | 0,0394 |
| insula R | 0,286 | 0,0463 |
| lateralorbitofrontal L | 0,3061 | 0,0381 |
| lateralorbitofrontal R | 0,2818 | 0,0473 |
| middletemporal L | 0,3189 | 0,0329 |
| middletemporal R | 0,3545 | 0,0258 |
| parahippocampal L | 0,3293 | 0,0277 |
| parsopercularis L | 0,356 | 0,0258 |
| parsopercularis R | 0,3326 | 0,027 |
| parsorbitalis L | 0,3831 | 0,0258 |
| parsorbitalis R | 0,4098 | 0,0258 |
| parstriangularis L | 0,3527 | 0,0258 |
| parstriangularis R | 0,3584 | 0,0258 |
| rostralanteriorcingulate R | 0,3335 | 0,027 |
| rostralmiddlefrontal L | 0,3392 | 0,027 |
| rostralmiddlefrontal R | 0,3664 | 0,0258 |
| superiorfrontal L | 0,2934 | 0,0418 |
| superiortemporal L | 0,3017 | 0,0387 |
| temporalpole L | 0,3164 | 0,0329 |
| temporalpole R | 0,302 | 0,0387 |
| transversetemporal R | 0,2858 | 0,0463 |

**Supplementary Table 6.** The present table enlists the *rho* and the FDR corrected *p-values* for each significant region in the node-wise correlation in the patients group between the exponent value and the number of antiseizure medications.

| **Exponent – Imm-recall RAVLT Spearman correlation** | | |
| --- | --- | --- |
| **Regions** | **rho** | **p-values** |
| bankssts L | -0,4696 | 0,0207 |
| cuneus L | -0,4444 | 0,0207 |
| cuneus R | -0,4319 | 0,0207 |
| entorhinal L | -0,3938 | 0,0347 |
| frontalpole L | -0,4428 | 0,0207 |
| fusiform L | -0,4405 | 0,0207 |
| inferiorparietal R | -0,3589 | 0,0406 |
| inferiortemporal L | -0,3549 | 0,0407 |
| insula L | -0,3747 | 0,0372 |
| lateraloccipital L | -0,4043 | 0,0347 |
| lateraloccipital R | -0,3851 | 0,036 |
| lateralorbitofrontal L | -0,3925 | 0,0347 |
| lingual L | -0,3934 | 0,0347 |
| lingual R | -0,3566 | 0,0407 |
| medialorbitofrontal R | -0,3725 | 0,0372 |
| middletemporal L | -0,3677 | 0,0372 |
| parsopercularis L | -0,4796 | 0,0207 |
| parsorbitalis L | -0,4945 | 0,0207 |
| parstriangularis L | -0,4419 | 0,0207 |
| pericalcarine L | -0,394 | 0,0347 |
| pericalcarine R | -0,4347 | 0,0207 |
| precuneus L | -0,3737 | 0,0372 |
| precuneus R | -0,3877 | 0,036 |
| rostralanteriorcingulate R | -0,3588 | 0,0406 |
| rostralmiddlefrontal L | -0,3699 | 0,0372 |
| rostralmiddlefrontal R | -0,4327 | 0,0207 |
| superiortemporal L | -0,367 | 0,0372 |
| supramarginal L | -0,3793 | 0,0372 |

**Supplementary Table 7.** The present table enlists the *rho* and the FDR corrected *p-values* for each significant region in the node-wise correlation in the patients group between the exponent value and the immediate recall value of the Rey Auditory Verbal Learning Test.
